# Combined chelation therapy in thalassemia major for the treatment of severe myocardial siderosis with left ventricular dysfunction

**DOI:** 10.1186/1532-429X-10-12

**Published:** 2008-02-25

**Authors:** Mark A Tanner, Renzo Galanello, Carlo Dessi, Gillian C Smith, Mark A Westwood, Annalisa Agus, Martina Pibiri, Sunil V Nair, J Malcolm Walker, Dudley J Pennell

**Affiliations:** 1 Cardiovascular Magnetic Resonance Unit, Royal Brompton Hospital, London, UK; 2 Ospedale Regionale per le Microcitemie, Cagliari, Italy; 3 Department of Cardiology, University College Hospital, London, UK

## Abstract

**Background:**

In thalassemia major (TM), severe cardiac siderosis can be treated by continuous parenteral deferoxamine, but poor compliance, complications and deaths occur. Combined chelation therapy with deferiprone and deferoxamine is effective for moderate myocardial siderosis, but has not been prospectively examined in severe myocardial siderosis.

**Methods:**

T2* cardiovascular magnetic resonance (CMR) was performed in 167 TM patients receiving standard subcutaneous deferoxamine monotherapy, and 22 had severe myocardial siderosis (T2* < 8 ms) with impaired left ventricular (LV) function. Fifteen of these patients received combination therapy with subcutaneous deferoxamine and oral deferiprone with CMR follow-up.

**Results:**

At baseline, deferoxamine was prescribed at 38 ± 10.2 mg/kg for 5.3 days/week, and deferiprone at 73.9 ± 4.0 mg/kg/day. All patients continued both deferiprone and deferoxamine for 12 months. There were no deaths or new cardiovascular complications. The myocardial T2* improved (5.7 ± 0.98 ms to 7.9 ± 2.47 ms; p = 0.010), with concomitant improvement in LV ejection fraction (51.2 ± 10.9% to 65.6 ± 6.7%; p < 0.001). Serum ferritin improved from 2057 (CV 7.6%) to 666 (CV 13.2%) μg/L (p < 0.001), and liver iron improved (liver T2*: 3.7 ± 2.9 ms to 10.8 ± 7.3 ms; p = 0.006).

**Conclusion:**

In patients with severe myocardial siderosis and impaired LV function, combined chelation therapy with subcutaneous deferoxamine and oral deferiprone reduces myocardial iron and improves cardiac function. This treatment is considerably less onerous for the patient than conventional high dose continuous subcutaneous or intravenous deferoxamine monotherapy, and may be considered as an alternative. Very prolonged tailored treatment with iron chelation is necessary to clear myocardial iron, and alterations in chelation must be guided by repeated myocardial T2* scans.

**Trial registration:**

This trial is registered as NCT00103753

## Introduction

Globally, there are at least 60,000 individuals born with thalassemia major (TM) each year [1]. Regular blood transfusions are mandatory for long term survival, but over a period of years these cause a secondary state of tissue iron overload. Myocardial iron deposition can result in cardiomyopathy, and heart failure remains the leading cause of death [2-4]. The introduction of the iron chelator deferoxamine greatly ameliorates the effects of iron toxicity, but long-term cardiac mortality has been very disappointing [3,5]. The ongoing deaths from cardiac iron loading may relate to inadequate compliance or genetic factors related to metal transporters not yet fully elucidated [6-8], but whatever the cause, there is strong evidence that long-term deferoxamine chelation does not effectively prevent myocardial siderosis in a majority of patients [9,10]. Deferiprone, the first approved oral chelator, has been shown in randomized controlled trials to be effective monotherapy at 100 mg/kg/day in treating mild to moderately severe myocardial iron loading (myocardial T2* 8–20 ms), significantly improving both myocardial iron and ejection fraction [11], and the combination of deferiprone at 75 mg/kg/day with deferoxamine is likewise effective [12]. However greater total iron clearance is seen with combined treatment [13-15], which suggests that it might be useful for severe myocardial siderosis (T2* < 10 ms).

The conventional treatment at many centers for severe myocardial siderosis with heart failure is long-term, continuous, high-dose intravenous deferoxamine. Several small studies have confirmed that this approach is effective, and reversal of cardiomyopathy is possible [16,17]. However, deaths still occur, complications such as infection and thrombosis are common, and poor compliance with these demanding regimes is problematic [16]. Combined chelation therapy in this situation might be effective, yet prospective trials examining the treatment of severe cardiac siderosis are lacking. Cardiovascular magnetic resonance (CMR) permits highly reproducible measurements of myocardial iron loading (T2*) [18,19], and ventricular function [20,21], making this modality ideally suited to assessing cardiovascular response to chelation treatments in TM [9-12]. Therefore, we prospectively examined the effects of combination therapy in TM patients with severe myocardial siderosis.

## Methods

### Study protocol

As part of a previously reported randomized controlled trial investigating the combined therapy of deferoxamine and deferiprone in mild-moderate myocardial siderosis [12], 167 adult patients (75 males, 30 ± 5.3 years) with TM were screened for quantification of myocardial iron loading using myocardial T2* [10]. These subjects were recruited from 12 centers in Sardinia, Italy. The inclusion criteria for patient screening were: diagnosis of beta-thalassemia major currently maintained on subcutaneous deferoxamine monotherapy; age >18 years; maintaining pre-transfusion hemoglobin > 9 g/dL. Exclusion criteria were: Patients who had previously received deferiprone for a total of more than 6 months over the last 5 years; patients with previous reaction to deferiprone; neutropenia (ANC <1.5 × 10^9^/L) at screening; thrombocytopenia (<50 × 10^9^/L) at screening; liver enzymes > 3 times upper limit of normal; and implant incompatible with MR (such as pacemaker), or other condition making CMR impossible or inadvisable (*e.g*. claustrophobia).

Of the 167 patients screened, 108 (65%) had significant myocardial siderosis (T2* < 20 ms), of whom 22 (13%) had severe myocardial loading (T2* < 8 ms) [10]. Patients with severe cardiac siderosis (T2* < 8 ms) were not randomized into the randomized controlled trial in accord with the study protocol, and it was at the treating clinician's discretion to determine best clinical practice chelation therapy. The threshold of 8 ms was set in accord with practice at the time of approval of the protocol by the ethics committee, although a myocardial T2* of 10 ms is typically considered the contemporary threshold for defining severe myocardial iron loading. We prospectively studied those patients with severe myocardial siderosis who received combination chelation therapy (deferiprone plus deferoxamine). The study protocol was approved by Ethics committees in London and Cagliari. Patient information and consent forms were in Italian and all patients gave written informed consent. The authors had full access to the data and took responsibility for its integrity. All authors have read and agree to the manuscript as written.

### Study patients

Of the 22 patients with severe myocardial siderosis, 15 (9 female, 28.9 ± 4.8 years) received unblinded combination therapy according to locally developed protocols, and were followed prospectively. These 15 patients form the subject of this paper. All 15 patients (100%) had impaired left ventricular systolic function relative to published normal values for thalassemia patients without myocardial iron loading [22]. Of the remaining 7 subjects, 3 subjects received intensive monotherapy with deferoxamine, 1 patient received deferiprone monotherapy, and in 3 patients treatment was maintained at local centers outside the study and were not included. One patient who was maintained on deferoxamine monotherapy died of cirrhotic liver disease.

In the 15 study patients, 9 (60%) had a prior history of heart failure, and all 9 were taking cardiac medications (angiotensin converting enzyme (ACE) inhibitor ± diuretic). Two of these subjects were in decompensated congestive cardiac failure at the time of screening. At baseline, 1 of the patients with symptomatic heart failure was in atrial flutter. The remaining 14 subjects were in sinus rhythm. At baseline, all patients were being maintained on subcutaneous deferoxamine monotherapy (no patient was receiving deferiprone) with a mean dose of 38 mg/kg for 5.3 days/week (equivalent to 40.3 mg/kg/d for 5 days/week). This is comparable to the 40–50 mg/kg/day for 5 days/week from clinical recommendations for routine practice [23].

### Efficacy and safety assessments

A UK-based mobile CMR scanner (1.5 Tesla, Sonata, Siemens, Erlangen, Germany) was transported on 3 occasions to Cagliari, Sardinia. The 15 patients on combination therapy underwent CMR assessments at baseline, 6 and 12 months to assess myocardial and hepatic iron loading (T2*), left ventricular (LV) volumes, and ejection fraction (EF). Myocardial and hepatic T2* were assessed using the single breath-hold multi-echo technique [24], with bright blood [25]. T2* analysis was performed using Thalassemia-Tools (a plug-in of CMRtools, Cardiovascular Imaging Solutions, London, UK) as described previously [24]. Ventricular volumes were determined using steady state free precession cines, with contiguous short axis slices from base to apex [26]. Ventricular volumes were also analyzed using CMRtools. As LV volumes and mass vary with height and weight these were indexed to body surface area (BSA). Other laboratory measures included weekly full blood count (risk of agranulocytosis with deferiprone), serum ferritin (Abbott AXSYM System), B-type natriuretic peptide (BNP-Biosite Diagnostics Inc, San Diego, California), and liver function tests (alanine aminotransferase [ALT]).

### Statistical analysis

Trend analysis over time for continuous parameters with normal distribution was performed using repeated measures ANOVA. Where the ANOVA was significant, the changes in parameters between time points were further analyzed using Least Significant Difference post-hoc testing. The Shapiro-Wilk test of normality identified BNP and serum ferritin as having non-parametric distributions. Serum ferritin levels demonstrated a positively skewed distribution and were subsequently log transformed to achieve normality. In the case of BNP measurements analysis was performed by Kruskal-Wallis testing. T2* and other parameters did not deviate significantly from normal in this study. Summary data are presented as mean ± standard deviation (SD), except ferritin data which are presented using the geometric mean (the antilog of the mean in log scale) ± the coefficient of variation (CV, equivalent to the variance of the mean in log scale). Statistical analysis was performed using SPSS Version 10.0 (SPSS, Inc, Chicago, Illinois). A p-value of 0.05 was the threshold used for statistical significance.

## Results

### Patient Characterization

There were no subject withdrawals with all 15 patients continuing with the combined chelation therapy for the full 12 months duration of the study. Baseline patient characteristics are given in table 1. During the study period, patients received combined chelation therapy for a mean of 11.7 ± 1.6 months.

**Table 1:** 

Age (years)	28.9 ± 4.8
Gender	
Male (M)	6
Female (F)	9
Body Surface Area (m^2^)	1.56 ± 0.1
Deferoxamine dose (mg/kg)	38 ± 10.2
	5.3 days/week
CMR measures:	
Myocardial T2* (ms) [>20]	5.7 ± 1.0
Liver T2* (ms) [>19]	3.7 ± 2.9
LV end diastolic volume index (mL/m^2^) [M:45–152, F:54–121]	87.1 ± 31.6
LV end systolic volume index (mL/m^2^) [M:13–34, F:6–35]	44.4 ± 24.9
LV ejection fraction (%) [M > 59%; F > 63%]	51.2 ± 10.9
Blood measures:	
Transfusional red blood cell input (mL/kg/year)	155.1 ± 40.9
Mean year hemoglobin (g/dL)	11.0 ± 1.2
Hepatitis C positive	
Yes	14
No	1
Biochemistry	
Serum ferritin (μg/L) [M: 5–104, F: 4–254 ]	2057 ± 7.6
Alanine aminotransferase (IU/L) [5-35]	53.4 ± 34.8
BNP (pmol/L) [<100]	26.0 (9.8–758)
Serum creatinine (mg/dL) [0.6–1.2]	0.68 ± 0.19

### Iron chelation

The dose of deferoxamine prescribed at baseline was 38.0 ± 10.2 mg/kg for 5.3 days/week. At the end of 12 months the prescribed dose had been reduced to 20.3 ± 10.9 for 4.5 days/week (p < 0.001). This dosing decrease reflects the standard clinical practice of dose reduction of deferoxamine in subjects with substantial falls in serum ferritin to minimize toxicity. The starting dose of deferiprone prescribed was 73.9 ± 4.0 mg/kg/day for 7 days per week. At 12 months, this had been decreased to 65.7 ± 10.7 mg/kg/day (p = 0.01), with dose reduction necessary in some patients due to mild adverse events (gastro-intestinal symptoms). All patients received both deferiprone and deferoxamine throughout the study period.

### Myocardial and hepatic iron

**Figure 1:**
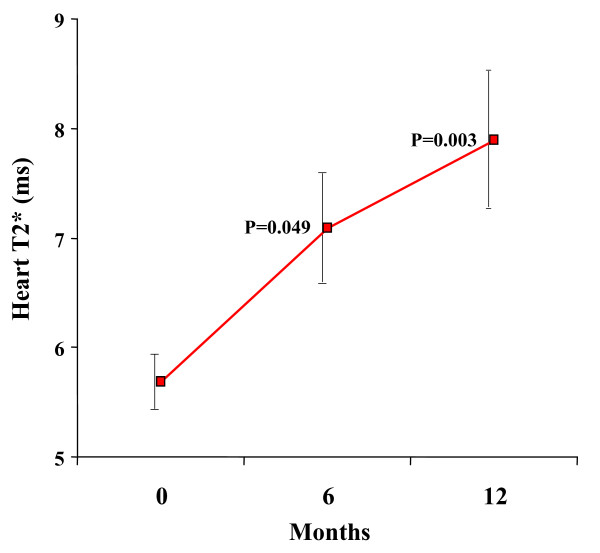
Myocardial T2* improved significantly between baseline and 12 months. Standard error bars are shown. The p values shown are post-hoc analyses for 0–6 months, and 0–12 months.

**Figure 2:**
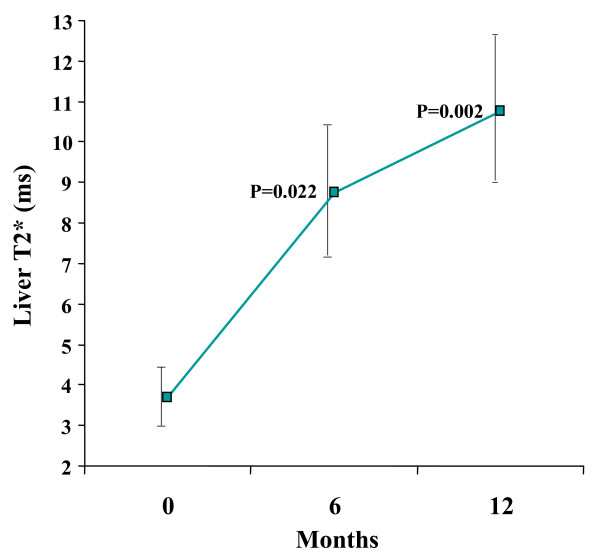
Liver T2* improved significantly between baseline and 12 months. Standard error bars are shown. The p values shown are post-hoc analyses for 0–6 months, and 0–12 months.

Myocardial T2* improved significantly from baseline 5.7 ± 0.98 ms to 7.1 ± 1.96 ms at 6 months, and 7.9 ± 2.47 ms at 12 months (ANOVA p = 0.010; figure 1, table 2). Liver T2* improved significantly from a baseline of 3.7 ± 2.9 ms to 8.8 ± 6.4 ms at 6 months, and 10.8 ± 7.3 ms at 12 months (ANOVA p = 0.006; figure 2).

**Table 2:** 

**Subject**	**Sex**	**Baseline NYHA Heart Failure Class**	**BASELINE**		**12 MONTHS**	
			Myocardial T2* (ms)	LVEF (%)	Myocardial T2* (ms)	LVEF (%)

**1**	F	**I**	5.2	59	6.2	70
**2**	M	**I**	6.0	28	6.4	57
**3**	M	**I**	6.5	50	10.0	68
**4**	M	**I**	6.4	42	9.2	63
**5**	M	**II**	5.6	30	6.1	47
**6**	M	**I**	5.0	57	5.4	73
**7**	F	**I**	4.8	60	5.5	74
**8**	F	**III**	5.3	43	7.6	67
**9**	F	**I**	5.0	54	8.1	67
**10**	M	**I**	5.7	58	6.6	67
**11**	F	**I**	5.4	49	10.4	66
**12**	F	**I**	7.3	61	11.5	70
**13**	F	**I**	3.7	61	4.8	61
**14**	F	**I**	7.4	60	13.2	65
**15**	F	**I**	6.1	56	7.1	69

### Cardiac function and volumes

**Figure 3:**
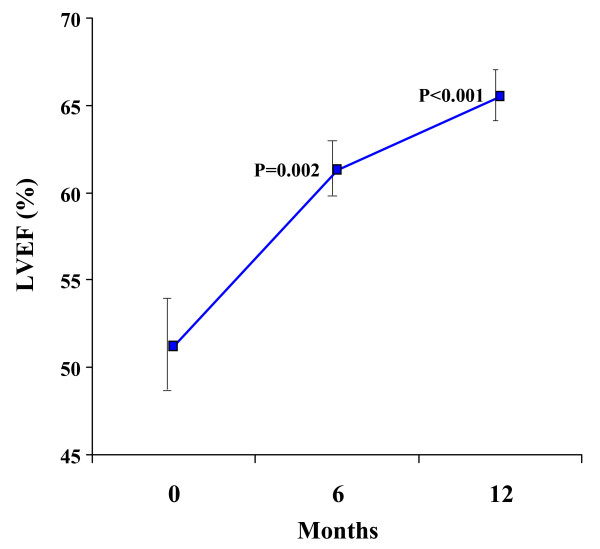
**There was a significant improvement in LV ejection fraction over 12 months.** Standard error bars are shown. The p values shown are post-hoc analyses for 0–6 months, and 0–12 months.

**Figure 4:**
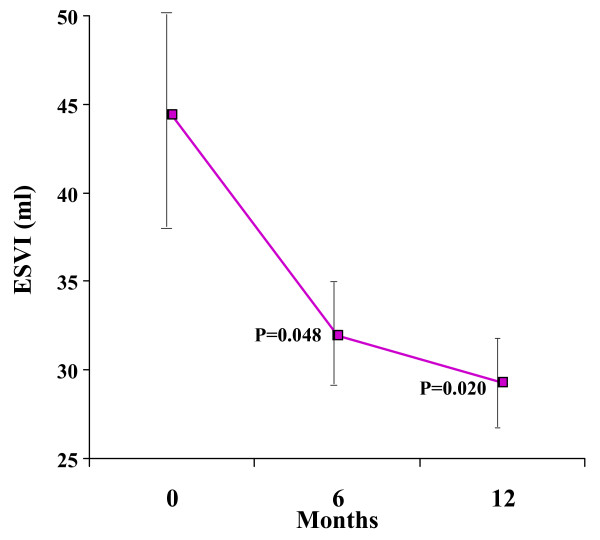
**LV end-systolic volume index decreased significantly over 12 months.** Standard error bars are shown. The p values shown are post-hoc analyses for 0–6 months, and 0–12 months.

There was a significant improvement in LVEF over 12 months, increasing from 51.2 ± 10.9% at baseline to 65.6 ± 6.7% at 12 months (ANOVA p < 0.001, figure 3, Table 2). End systolic volume index (ESVI) decreased significantly, from a baseline value of 44.4 ± 24.9 mL/m^2 ^to 29.3 ± 11.1 mL/m^2 ^at 12 months (ANOVA p = 0.043, figure 4). There was a non-significant change in end diastolic volume index (EDVI) from a baseline of 87.1 ± 31.6 mL/m^2 ^to 83.5 ± 17.4 mL/m^2 ^at 12 months (p = 0.72). LV mass index did not change significantly (baseline 85.2 ± 14.2 g/m^2^; 12 months, 83.9 ± 14.4 g/m^2^, p = 0.93).

### Serum Ferritin

**Figure 5:**
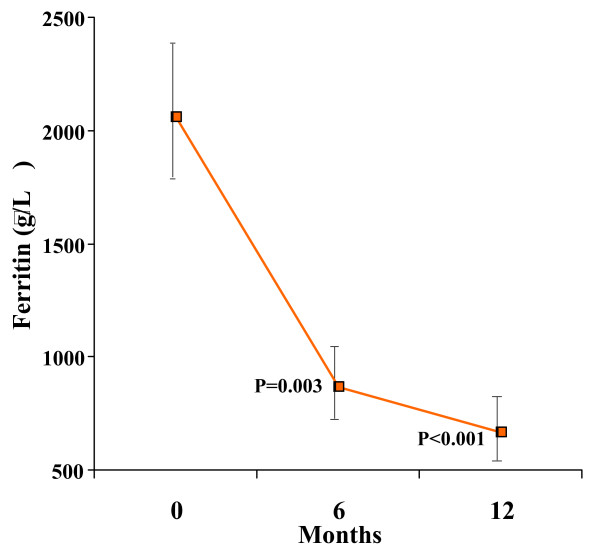
**There was a significant reduction in serum ferritin over 12 months.** The vertical axis shows the geometric mean of ferritin. Standard error bars are shown. The p values shown are post-hoc analyses for 0–6 months, and 0–12 months.

There was a significant reduction in ferritin, from 2057 μg/L (CV 7.6%) at baseline to 666 μg/L (CV 13.2%) at 12 months (ANOVA p < 0.001; figure 5).

### BNP

Only 2 patients at baseline had elevated BNP levels (>100 pmol/l), and both these patients were in overt cardiac failure. The BNP levels normalized within 6 months. Over 12 months there was no significant change in BNP in the whole group (26.0 to 21.6 pmol/l over 12 months, p = 0.56).

### Cardiac complications and medication

At baseline, 2 patients were in overt cardiac failure (subjects 5 and 8, table 2). One of these patients was also in atrial flutter. This patient was briefly hospitalized for inotropic support (dopamine), diuresis, and commencement of combination therapy, and subsequently made a steady recovery, reverting to sinus rhythm with no further cardiac complications during the study period. The other patient in heart failure responded to diuresis and continued ACE inhibition, and did not require inotropic support. There were no other cardiac complications in the remaining 13 patients. During the trial period no new cardiac medications were commenced in any of these 13 patients.

### Adverse events

There were no episodes of neutropenia or agranulocytosis. The most commonly reported side effects were mild gastro-intestinal symptoms (4/15 (27%)). There was one case of mild transient arthralgia, and one case of transient elevation of ALT (deferiprone was continued).

## Discussion

Heart failure secondary to myocardial iron loading remains the single most important cause of death in patients with TM [2,3]. It is well recognized that LV dysfunction and symptomatic heart failure occur at an advanced stage of cardiac siderosis, at which point treatment can be difficult and there is a poor prognosis [27,28]. Non-continuous deferoxamine monotherapy appears to be inadequate in such patients [29,30]. Intensive, continuous intravenous deferoxamine is probably more effective, and reverses cardiomyopathy in many, but not all cases [16,17], and many centers consider this (or continuous subcutaneous infusion [31]) to be the standard of care. Combined therapy with deferoxamine and deferiprone has been demonstrated to be of clear benefit in the treatment of cardiac siderosis in TM [12,13,32,33], but the role of combined therapy in the management of severe cardiac siderosis has not been specifically addressed beyond a few case reports [34-36] yet it has potential advantages over standard intensive intravenous chelation. Firstly, the presence of a long-term intravenous central catheter is not required which obviates the attendant risks (including infection and thrombosis). Secondly, the risk of deferoxamine toxicity might be reduced through the lower doses used in combined therapy. Thirdly, the use of intermittent subcutaneous deferoxamine therapy is less disruptive to patients and could confer improved compliance, which is considered a key factor in improving mortality in TM [16,37]. Finally, there is substantial evidence to suggest that deferiprone has superior cardioprotective effects [4,11,38,39], which remain to be fully explained, but anti-oxidant effects and influence on iron transport mechanisms are possibilities [6-8].

This study demonstrates that combined therapy in patients with severe cardiac siderosis results in large and significant improvements in myocardial iron loading and ventricular function. In all cases bar one, an improvement in myocardial T2* was coupled with an improvement in ejection fraction. In this one case, the apparent lack of improvement in cardiac function might reflect unknown biological factors, or measurement error. Despite comparable chelation regimes, some subjects showed far greater improvements in myocardial iron clearance and cardiac function compared to others. Although not formally assessed in this study, the degree of compliance to the prescribed chelation dose is likely to account for the majority of these observed differences. Other factors that might play a role in determining response to therapy include individual variation in chelation metabolism, or genetic factors involved in the regulation of myocardial iron metabolism. Clearance of iron from the heart is slow with all treatments in severe cardiac siderosis however, and treatment for 12 months is clearly inadequate to clear the cardiac iron, indicating that changes in chelation tailored to the cardiac siderosis must be guided by repeated cardiac T2* measurements. It would appear from the current trial that treatment of these patients would be necessary at the same chelation intensity for approximately 3–4 years to raise the T2* above 20 ms (assuming that the rate of clearance is constant). During this period the liver iron and ferritin will become low, and one clear advantage of the combination regime is that the likelihood of adverse effects of deferoxamine in these circumstances can be ameliorated through appropriate dose reduction (20.3 mg/kg for 4.5 days per week at the end of 12 months treatment in this study).

The rate of clearance of myocardial iron in the current trial of combination therapy (5.7 to 7.9 ms) was similar to that reported in a previous prospective study by Anderson who reported the changes in myocardial T2* in patients presenting with heart failure due to cardiac siderosis in response to high dose 24 hour per day intravenous deferoxamine for 12 months (5.1 to 8.1 ms) [17]. In both these studies, there were impressive improvements in ejection fraction (current study +14.4%; Anderson's study +11%) from a similar baseline (current study 51.2%; Anderson's study 52.0%). This emphasizes the relatively high value of ejection fraction at which patients with TM develop heart failure compared to patients with coronary disease and cardiomyopathy. Recently, the normal reference range for LV volumes and function in TM *without *myocardial iron have been determined by CMR [22]. An adaptive response to their chronic anemic state and other factors produces a hyperdynamic circulation, resulting in significantly different LV parameters as compared with normal controls. The mean LV ejection fraction is therefore significantly increased in TM patients, with the mean measured by CMR for women being 75.1 ± 5.9%, and men 71.0% ± 6.1%. These are 6–8% absolute units higher than normal subjects and indicate that TM patients have impaired LV function at higher values of LV ejection fraction than previously thought. Improvements in ejection fraction were mediated via a reduction in end systolic volume, with end diastolic volume not being significantly changed. This reflects no change in volume loading, but an improvement in contractility.

These results also demonstrate that following the addition of deferiprone, the total weekly deferoxamine dose and infusion frequency can be significantly reduced without compromising myocardial iron loading and function. The incidence of adverse effects was low and consistent with prior studies of these chelators. A recent prospective study has demonstrated that twice weekly deferoxamine infusions in conjunction with deferiprone five times per week, shows similar efficacy to standard deferoxamine monotherapy in removing iron [40]. A reduction in intensity of deferoxamine chelation is likely to confer considerable benefits to the patient. In Anderson's study, where much higher doses of continuous deferoxamine were used (mean dose 46 mg/kg/day, 7 days/week), neuro-toxicity was documented in two-thirds of patients. No cases of deferoxamine toxicity were reported in this study. The reduced dose frequency seen in the current study (4.5 days/week at 12 months) is likely to be more convenient to patients and potentially improve compliance.

This study addresses a number of other issues regarding the management of cardiac siderosis in TM. CMR techniques have recently illuminated the weak relation between liver iron/ferritin, and myocardial siderosis [10,41,42]. This has challenged existing opinion that survival without cardiac disease is good where serum ferritin levels and liver iron remain below 2500 μg/l and 15 mg/g/dry weight, respectively [2,43-45]. The 3-year mean ferritin levels, and baseline liver iron concentrations of this cohort are given in table 3. The vast majority of these patients with severe cardiac siderosis would have been considered at low risk of cardiac complications on the basis of traditional static ferritin and liver iron concentration based thresholds. Yet all these subjects demonstrated LV dysfunction at baseline. Both subjects with decompensated heart failure had 3 year mean ferritin levels <2500 μg/L, and baseline liver iron concentration <15 mg/g/dry weight. These findings highlight the difficulties in using such surrogate parameters to guide cardiac management.

**Table 3:** 

Subject	Mean 3 year ferritin (μg/L)	Liver T2* (ms)	Liver Iron Concentration (mg/g/dry weight)
**1**	2297	1.5	9.3
**2**	1135	3.0	4.4
**3**	1273	1.9	7.1
**4**	1211	8.4	1.5
**5**	2342	1.9	7.1
**6**	1400	1.7	8.2
**7**	3667	0.95	15.2
**8**	2442	1.2	11.8
**9**	1867	4.1	3.2
**10**	1957	3.9	3.4
**11**	500	1.6	8.6
**12**	967	11.2	1.1
**13**	2835	3.2	4.2
**14**	1250	5.0	2.6
**15**	1536	5.9	2.2

BNP has been found to have diagnostic and prognostic use in non-siderotic heart failure [46,47], but data regarding its application in thalassemia is sparse [10]. In this cohort, only the 2 subjects in overt cardiac failure had abnormal BNP levels. This suggests that BNP levels become raised only at a very advanced stage, and are therefore unlikely to contribute significantly to the pre-clinical identification of siderotic cardiomyopathy. The lack of significant change in BNP over 12 months (in the whole group) despite considerable improvements in cardiac function and cardiac siderosis also suggests little role for serial BNP measurements in these patients. It is possible that an explanation for this finding is that BNP secretion is impaired in cardiac siderosis (cardiac endocrinopathy).

## Limitations

This study was limited by the relatively low number of patients and its non-comparative nature. This precludes definitive conclusions about the relative efficacy as compared to continuous intravenous deferoxamine therapy for the treatment of severe cardiac siderosis. However, the findings of improved myocardial iron loading and cardiac function mimic those recently demonstrated in the larger randomized controlled trial in patients with mild-moderately severe cardiac siderosis [12], suggesting that combination therapy is also effective in this patient group.

## Conclusion

Combined chelation therapy in patients with severe cardiac siderosis is well tolerated, and effective in removing myocardial iron and improving ventricular function. These findings suggest that this therapy may be considered as an alternative to continuous subcutaneous or intravenous deferoxamine in the management of these patients.

## Competing interests

Professor Pennell had full access to all the data in the study and had final responsibility for the decision to submit for publication. The authors are solely responsible for the conduct, data storage, data analysis and reporting of this trial. Professor Pennell is a consultant to, has received speaker's honoraria and research support from, and has participated in chelation drug research with Apotex. He is also a consultant to, and is participating in, chelation drug research with Novartis. He is a consultant to Siemens Medical Solutions, and a director of Cardiovascular Imaging Solutions. Professor Galanello has received speaker's honoraria and research support from Apotex and Novartis. Ms Smith is a consultant to, and is participating in chelation drug research with Novartis. Dr Walker has received research support from Novartis and speaker's honoraria from Apotex. Dr Westwood has received speaker's honoraria from Apotex. Dr Nair was supported by the British Heart Foundation. The other authors have no interests to declare.

## Authors' contributions

All authors have seen and approved the final version of the manuscript. DP co-designed and coordinated the study, managed the research, and co-wrote the manuscript. RG co-designed and coordinated the study, and managed the trial patients. MT coordinated the study, acquired and analyzed the CMR data, and co-wrote the manuscript. CD, MP, and AA were involved in the management of the trial patients. GS acquired and analyzed the CMR data. MW was involved in the study design and data acquisition. SN was involved in data acquisition and analysis. MW was involved in the study design, data acquisition and analysis.

## References

[B1] Weatherall DJ, Clegg JB (2001). The thalassaemia syndromes.

[B2] Olivieri NF, Nathan DG, MacMillan JH, Wayne AS, Liu PP, McGee A, Martin M, Koren G, Cohen AR (1994). Survival in medically treated patients with homozygous beta-thalassemia. N Engl J Med.

[B3] Borgna-Pignatti C, Rugolotto S, De Stefano P, Zhao H, Cappellini MD, Del Vecchio GC, Romeo MA, Forni GL, Gamberini MR, Ghilardi R, Piga A, Cnaan A (2004). Survival and complications in patients with thalassemia major treated with transfusion and deferoxamine. Haematologica.

[B4] Telfer P, Coen PG, Christou S, Hadjigavriel M, Kolnakou A, Pangalou E, Pavlides N, Psiloines M, Simamonian K, Skordos G, Sitarou M, Angastiniotis M (2006). Survival of medically treated thalassemia patients in Cyprus. Trends and risk factors over the period 1980–2004. Haematologica.

[B5] Modell B, Khan M, Darlison M (2000). Survival in beta thalassaemia major in the UK: Data from the UK Thalassaemia Register. Lancet.

[B6] Oudit GY, Sun H, Trivieri MG, Koch SE, Dawood F, Ackerley C, Yazdanpanah M, Wilson GJ, Schwartz A, Liu PP, Backx PH (2003). L-type Ca2+ channels provide a major pathway for iron entry into cardiomyocytes in iron-overload cardiomyopathy. Nat Med.

[B7] Oudit GY, Trivieri MG, Khaper N, Liu PP, Backx PH (2006). Role of L-type Ca2+ channels in iron transport and iron-overload cardiomyopathy. J Mol Med.

[B8] Ludwiczek S, Theurl I, Muckenthaler MU, Jakab M, Mair SM, Theurl M, Kiss J, Paulmichl M, Hentze MW, Ritter M, Weiss G (2007). Ca2+ channel blockers reverse iron overload by a new mechanism via divalent metal transporter-1. Nat Med.

[B9] Anderson LJ, Wonke B, Prescott E, Holden S, Walker JM, Pennell DJ (2002). Comparison of effects of oral deferiprone and subcutaneous desferrioxamine on myocardial iron levels and ventricular function in beta thalassemia. Lancet.

[B10] Tanner MA, Galanello R, Dessi C, Westwood MA, Smith GC, Nair SV, Anderson LJ, Walker JM, Pennell DJ (2006). Myocardial iron loading in patients with thalassaemia major on deferoxamine chelation. J Cardiovasc Magn Reson.

[B11] Pennell DJ, Berdoukas V, Karagiorga M, Ladis V, Piga A, Aessopos A, Gotsis S, Tanner MA, Smith GC, Westwood MA, Wonke B, Galanello R (2006). Randomized controlled trial of the effect of deferiprone or deferoxamine on myocardial iron and function in beta-thalassemia major. Blood.

[B12] Tanner MA, Galanello R, Dessi C, Smith GC, Westwood MA, Agus A, Roughton M, Assomull R, Nair SV, Walker JM, Pennell DJ (2007). A randomized, placebo-controlled, double-blind trial of the effect of combined therapy with deferoxamine and deferiprone on myocardial iron in thalassemia major using cardiovascular magnetic resonance. Circulation.

[B13] Origa R, Bina P, Agus A, Crobu G, Defraia E, Dessi C, Leoni G, Muroni PP, Galanello R (2005). Combined therapy with deferiprone and desferrioxamine in thalassemia major. Haematologica.

[B14] Wonke B, Wright C, Hoffbrand AV (1998). Combined therapy with deferiprone and desferrioxamine. Br J Haematol.

[B15] Link G, Konijn AM, Breuer W, Cabantchik ZI, Hershko C (2001). Exploring the "iron shuttle" hypothesis in chelation therapy: effects of combined deferoxamine and deferiprone treatment in hypertransfused rats with labeled iron stores and in iron-loaded rat heart cells in culture. J Lab Clin Med.

[B16] Davis BA, Porter JB (2000). Long-term outcome of continuous 24-hour deferoxamine infusion via indwelling intravenous catheters in high-risk beta-thalassemia. Blood.

[B17] Anderson LJ, Westwood MA, Holden S, Davis B, Prescott E, Wonke B, Porter JB, Walker JM, Pennell DJ (2004). Myocardial iron clearance during reversal of siderotic cardiomyopathy with intravenous desferrioxamine: a prospective study using T2* cardiovascular magnetic resonance. Br J Haematol.

[B18] Westwood M, Anderson LJ, Firmin DN, Gatehouse PD, Lorenz CH, Wonke B, Pennell DJ (2003). Interscanner reproducibility of cardiovascular magnetic resonance in the early diagnosis of myocardial iron overload. J Magn Reson Imaging.

[B19] Tanner MA, He T, Westwood MA, Firmin DN, Pennell DJ (2006). Thalassemia International Federation Heart T2* Investigators. Multi-center validation of the transferability of the magnetic resonance T2* technique for the quantification of tissue iron. Haematologica.

[B20] Maceira AM, Prasad SK, Khan M, Pennell DJ (2006). Normalized left ventricular systolic and diastolic function by steady state free precession cardiovascular magnetic resonance. J Cardiovasc Magn Reson.

[B21] Maceira AM, Prasad SK, Khan M, Pennell DJ (2006). Reference right ventricular systolic and diastolic function normalized to age, gender and body surface area from steady-state free precession cardiovascular magnetic resonance. Eur Heart J.

[B22] Westwood MA, Anderson LJ, Maceira AM, Shah FT, Prescott E, Porter JB, Wonke B, Walker JM, Pennell DJ (2007). Normalized left ventricular volumes and function in thalassemia major patients with normal myocardial iron. J Magn Reson Imaging.

[B23] Porter JB (2001). Practical management of iron overload. Br J Haematol.

[B24] Westwood M, Anderson LJ, Firmin DN, Gatehouse PD, Charrier CC, Wonke B, Pennell DJ (2003). A single breath-hold multiecho T2* cardiovascular magnetic resonance technique for diagnosis of myocardial iron overload. J Magn Reson Imaging.

[B25] He T, Gatehouse PD, Kirk P, Tanner MA, Smith GC, Keegan J, Mohiaddin RH, Pennell DJ, Firmin DN (2007). Black-blood T2* technique for myocardial iron measurement in thalassemia. J Magn Reson Imaging.

[B26] Pennell DJ, Zipes DP, Libby P, Bonow RO, Braunwald E (2005). Cardiovascular Magnetic Resonance. Braunwald's Heart Disease: A textbook of cardiovascular medicine.

[B27] Olivieri NF, Snider MA, Nathan DG (1995). Survival following the onset of iron-induced cardiac disease in thalassaemia major. Blood (Abstract).

[B28] Jessup M, Manno CS (1998). Diagnosis and management of iron-induced heart disease in Cooley's anemia. Ann N Y Acad Sci.

[B29] Tamary H, Goshen J, Carmi D, Yaniv I, Kaplinsky C, Cohen IJ, Zaizov R (1994). Long-term intravenous deferoxamine treatment for noncompliant transfusion-dependent beta-thalassemia patients. Isr J Med Sci.

[B30] Cohen AR, Martin M, Schwartz E (1990). Current treatment of Cooley's anemia. Intravenous chelation therapy. Ann N Y Acad Sci.

[B31] Davis BA, O'Sullivan C, Jarritt PH, Porter JB (2004). Value of sequential monitoring of left ventricular ejection fraction in the management of thalassemia major. Blood.

[B32] Origa R, Bina P, Agus A, Crobu G, Defraia E, Dessi C, Leoni G, Muroni PP, Galanello R (2005). Combined therapy with deferiprone and desferrioxamine in thalassemia major. Haematologica.

[B33] Kattamis K, Ladis V, Berdousi H, Kelekis NL, Alexopoulou E, Papasotiriou I, Drakaki K, Kaloumenou I, Galani A, Kattamis C (2006). Iron chelation treatment with combined therapy with deferiprone and deferioxamine: a 12-month trial. Blood Cells Mol Dis.

[B34] Wu KH, Chang JS, Tsai CH, Peng CT (2004). Combined therapy with deferiprone and desferrioxamine successfully regresses severe heart failure in patients with beta-thalassaemia major. Ann Hematol.

[B35] Tsironi M, Deftereos S, Andriopoulos P, Farmakis D, Meletis J, Aessopos A (2005). reversal of heart failure in thalassaemia major by combined chelation therapy: a case report. Eur J Haematol.

[B36] Porcu M, Landis N, Salis S, Corda M, Orru P, Serra E, Usai B, Matta G, Galanello R (2007). Effects of combined deferiprone and desferrioxamine iron chelating therapy in beta-thalassemia major end-stage heart failure: a case report. Eur J Heart Fail.

[B37] Piga A, Longo F, Consolati A, De Leo A, Carmellino L (1997). Mortality and morbidity in thalassaemia with conventional treatment. In Proceedings of the third international conference on bone marrow transplantation in thalassaemia. Bone Marrow Transplantation.

[B38] Piga A, Caglioti C, Fogliacco E, Tricta F (2003). Comparative effects of deferiprone and deferoxamine on survival and cardiac disease in patients with thalassaemia major: a retrospective analysis. Haematologica.

[B39] Borgna-Pignatti C, Cappellini MD, De Stefano P, Del Vecchio GC, Forni GL, Gamberini MR, Ghilardi R, Piga A, Romeo MA, Zhao H, Cnaan A (2006). Cardiac morbidity and mortality in deferoxamine- or deferiprone-treated patients with thalassemia major. Blood.

[B40] Galanello R, Kattamis A, Piga A, Fischer R, Leoni G, Ladis V, Voi V, Lund U, Tricta F (2006). A prospective randomized controlled trial on the safety and efficacy of alternating deferoxamine and deferiprone in the treatment of iron overload in patients with thalassemia. Haematologica.

[B41] Anderson LJ, Holden S, Davies B, Prescott E, Charrier C, Bunce NH, Firmin DN, Porter JB, Wonke B, Walker JM, Pennell DJ (2001). Cardiovascular T2* (T2 star) magnetic resonance for the early diagnosis of myocardial iron overload. Eur Heart J.

[B42] Wood JC, Tyszka JM, Carson S, Nelson MD, Coates TD (2004). Myocardial iron loading in transfusion-dependent thalassemia and sickle cell disease. Blood.

[B43] Brittenham GM, Griffith PM, Nienhuis AW, McLaren CE, Young NS, Tucker EE, Allen CJ, Farrell DE, Harris JW (1994). Efficacy of deferoxamine in preventing complications of iron overload in patients with thalassemia major. N Engl J Med.

[B44] Kolnagu A, Econoimides C, Eracleous E, Kontoghiorghes GJ (2006). Low serum ferritin levels are misleading for detecting cardiac iron overload and increase the risk of cardiomyopathy in thalassaemia patients. The importance of cardiac iron overload monitoring using magnetic resonance imaging T2 and T2*. Hemoglobin.

[B45] Anderson LJ, Westwood MA, Prescott E, Walker JM, Pennell DJ, Wonke B (2006). Development of thalassemic cardiomyopathy despite low liver iron levels and meticulous compliance to desferrioxamine. Acta Haematol.

[B46] Maisel AS, Krishnaswany P, Nowak RM, McCord J, Hollander JE, Duc P, Omland T, Storrow AB, Abraham WT, Wu AH, Clopton P, Steg PG, Westheim A, Knudsen CW, Perez A, Kazanegra R, Herrmann HC, McCullough P (2002). Rapid measurement of B-type natriuretic peptide in the emergency diagnosis of heart failure. N Engl J Med.

[B47] Wang TJ, Larson MG, Levy D, Benjamin EJ, Leip EP, Omland T, Wolf PA, Vasan RS (2004). Plasma natriuretic peptide levels and the risk of cardiovascular events and deaths. N Engl J Med.

